# Fast Algorithms for Fitting Active Appearance Models to Unconstrained Images

**DOI:** 10.1007/s11263-016-0950-1

**Published:** 2016-09-24

**Authors:** Georgios Tzimiropoulos, Maja Pantic

**Affiliations:** 1 School of Computer Science, University of Nottingham, Nottingham, UK; 2 Department of Computing, Imperial College London, London, UK; 3 Faculty of Electrical Engineering, Mathematics and Computer Science, University of Twente, Enschede, The Netherlands

**Keywords:** Active Appearance Models, Face alignment, In-the-wild

## Abstract

Fitting algorithms for Active Appearance Models (AAMs) are usually considered to be robust but slow or fast but less able to generalize well to unseen variations. In this paper, we look into AAM fitting algorithms and make the following orthogonal contributions: We present a simple “project-out” optimization framework that unifies and revises the most well-known optimization problems and solutions in AAMs. Based on this framework, we describe robust simultaneous AAM fitting algorithms the complexity of which is not prohibitive for current systems. We then go on one step further and propose a new approximate project-out AAM fitting algorithm which we coin Extended Project-Out Inverse Compositional (E-POIC). In contrast to current algorithms, E-POIC is both efficient and robust. Next, we describe a part-based AAM employing a translational motion model, which results in superior fitting and convergence properties. We also show that the proposed AAMs, when trained “in-the-wild” using SIFT descriptors, perform surprisingly well even for the case of unseen unconstrained images. Via a number of experiments on unconstrained human and animal face databases, we show that our combined contributions largely bridge the gap between exact and current approximate methods for AAM fitting and perform comparably with state-of-the-art face alignment systems.

## Introduction

Pioneered by Cootes et al. (2001) and revisited by Matthews and Baker (2004), Active Appearance Models (AAMs) have been around in computer vision research for more than 15 years. They are statistical models of shape and appearance that can generate instances of a specific object class (e.g. faces) given a small number of model parameters. Fitting an AAM to a new image entails estimating the model parameters so that the model instance and the given image are *“close enough”* typically in a least-squares sense. Recovering the shape parameters is important because it implies that the location of a set of landmarks (or fiducial points) has been detected in the given image. Landmark localization is of fundamental significance in many computer vision problems like face and medical image analysis. Hence, fitting AAMs robustly to new images has been the focus of extensive research over the past years.

Fitting AMMs is an iterative process at each iteration of which an update of the current model parameters is estimated. Typically, the update is a function of the error between the image and the model instance measured in the canonical reference frame of the model. There are two main lines of research for modeling this function. The first is to learn it via regression which was also the approach proposed in the original AAM paper (Cootes et al. 2001). Regression-based approaches are fast but approximate. For example in Cootes et al. (2001), the relationship between the error image and the update is assumed linear and independent of the current model parameters.

Of particular interest in this work is the second line of research for fitting AAMs through non-linear least-squares (Matthews and Baker 2004). In particular, AAM fitting is formulated as a Lucas–Kanade (LK) problem (Lucas et al. 1981) which can be solved iteratively using Gauss–Newton optimization. However, standard gradient descend algorithms when applied to AAM fitting are inefficient. This problem was addressed in the seminal work of Matthews and Baker (2004) which extends the LK algorithm and the appearance-based tracking framework of Hager and Belhumeur (1998) for the case of AAMs and deformable models. One of the major contributions of Matthews and Baker (2004) is the so-called Project-Out Inverse Compositional algorithm (POIC). The algorithm is coined project-out because it decouples shape from appearance and inverse compositional because the update is estimated in the model coordinate frame and then composed to the current estimate (this is in contrast to the standard LK algorithm in which the parameters are updated in a forward additive fashion). The combination results in an algorithm which is as efficient as regression-based approaches and is now considered the standard choice for fitting person-specific AAMs (i.e. AAMs for modeling a specific subject known in advance during training). Its main disadvantage is its limited capability of generalizing well to unseen variations, the most notable example of which is the case of generic AAMs (i.e. AAMs for modeling subjects not seen during training).

In contrast to POIC, the Simultaneous Inverse Compositional (SIC) algorithm (Baker et al. 2003) has been shown to perform robustly for the case of generic fitting (Gross et al. 2005). However, the computational cost of the algorithm is almost prohibitive for most applications. Let *n* and *m* denote the number of the shape and appearance parameters of the AAM. Then, the cost per iteration for SIC is $O((n+m)^2N)$, where *N* is the number of pixels in the reference frame. Note that the cost of POIC is only *O*(*nN*). For generic fitting $m \gg n$ and hence the huge difference in computational cost has either ruled out SIC from most papers/studies that depart from the person-specific case or made the authors resort in approximate solutions (please see (Saragih and Gocke 2009) for an example).

Some attempts to reduce the cost of SIC do exist but they are limited. An example is the Normalization algorithm (Gross et al. 2005). However, the performance of the Normalization algorithm has been reported to be closer to that of POIC rather than that of SIC. A second notable example is Papandreou and Maragos (2008) which applies the update rules originally proposed in Hager and Belhumeur (1998) to the problem of AAM fitting. The framework proposed in this paper is also based on Hager and Belhumeur (1998), but it extends it in several different ways (please see below for a summary of our contributions). Finally, other techniques for reducing the cost to some extent via pre-computations have been reported in Batur and Hayes (2005) and Netzell and Solem (2008).

### Summary of Contributions and Paper Roadmap

In this paper, we attempt to address two important questions in AAM literature:

1. Can AAMs provide fitting performance comparable to that of contemporary state-of-the-art algorithms for the difficult problem of fitting facial deformable models to unconstrained images (also known as face alignment *in-the-wild*)?
2. What is the relation between performance and computational efficiency? How efficient can exact algorithms be? More importantly, are there any inexact algorithms that are also robust?

In the remaining of this paper, we show that the answer to the above questions is positive, however multiple contributions/improvements in almost orthogonal directions are necessary in order to achieve both aims. In particular, our contributions include:

1. Two approximate but both robust and efficient fitting algorithms coined approximate Fast-SIC (aFast-SIC) and Extended POIC (E-POIC) with complexity $O((n+m)N + n^2N)$ and *O*(*nN*), respectively. We show that aFast-SIC has essentially the same fitting and convergence properties as SIC, and can be seen as a block coordinate descent algorithm for AAM fitting. Additionally, we show that E-POIC largely bridges the gap between SIC and POIC, and can be seen as a very fast regression-based approach to AAM fitting.
2. Robust training of AAMs. In particular, we propose a new direction for employing AAMs in unconstrained conditions by means of training AAMs in-the-wild, and provide justifications why such a training procedure is beneficial for AAM fitting.
3. Part-based AAMs combined with a translational motion model (as opposed to standard holistic AAMs with piece-wise affine warp). We show that our part-based AAM, coined Gauss–Newton Deformable Part Model (GN-DPM), is more robust and accurate and has superior convergence properties.
4. Robust features, in particular SIFT-based AAMs. Although creating such models is straightforward, there is also an associated computational overhead. To alleviate this problem, we show how significant computational reductions can be achieved by building a full model during training but then efficiently performing the optimization on a sparse grid during fitting.

Having summarized the individual contributions of our paper above, we are now ready to state the main result of our paper: Our part-based AAM (GN-DPM) built from SIFT features and fitted with the E-POIC algorithm has essentially the same fitting performance as the same model fitted with aFast-SIC. Although E-POIC requires a few more iterations to converge, it is very efficient (*O*(*nN*)) and almost two orders of magnitude faster per iteration than aFast-SIC. Finally, in terms of fitting accuracy, both algorithms are shown to achieve state-of-the-art performance, and sometimes superior to that of two state-of-the-art methods, namely SDM (Xiong and De la Torre 2013) and Chehra (Asthana et al. 2014). In addition to human faces, our results are also verified on a newly collected and very challenging animal face data set.

Following Sects. 2–4 which provide related work in face alignment and AAMs, the aforementioned contributions are described in the following sections:

1. In Sect. 5, we present a simple optimization framework for AAM fitting that unifies and revises the most well-known optimization problems and solutions in AAMs. Our framework derives and solves the optimization problem for Fast-SIC, a fast algorithm that is theoretically guaranteed to provide exactly the same updates per iterations as the ones provided by SIC, and describes a simple and approximate Fast-SIC algorithm coined aFast-SIC.
2. In Sect. 6, and based on the analysis of Sect. 5, we describe our approximate but both robust and efficient AAM fitting algorithm coined E-POIC.
3. In Sect. 7, we illustrate the benefits of the proposed training of AAMs in-the-wild.
4. In Sect. 8.1, we describe the proposed part-based AAM, coined GN-DPM, and show how this model can be fitted with Gauss–Newton optimization via a translational motion model.
5. In Sect. 9, we describe the efficient fitting of SIFT-based AAMs based on a weighted least-squares formulation.
6. In Sect. 10, we report experiments illustrating the fitting and convergence properties of all AAMs described in this work and provide comparisons with state-of-the-art.

Finally, we conclude in Sect. 11. Portions of this work appear in two conference publications, please see Tzimiropoulos and Pantic (2013) and Tzimiropoulos and Pantic (2014).

## State-of-the-Art in Face Alignment

The problem of face alignment has a long history in computer vision and a large number of approaches have been proposed to tackle it. Typically, faces are modelled as deformable objects which can vary in terms of shape and appearance. Much of early work revolved around the Active Shape Models (ASMs) and the AAMs (Cootes et al. 1995, 2001; Matthews and Baker 2004). In ASMs, facial shape is expressed as a linear combination of shape bases learned via principal component analysis (PCA), while appearance is modelled locally using (most commonly) discriminatively learned templates. In AAMs, shape is modelled as in ASMs but appearance is modelled globally using PCA in a canonical coordinate frame where shape variation has been removed. More recently, the focus has been shifted to the family of methods coined Constrained Local Models (CLMs) (Cristinacce and Cootes 2008; Lucey et al. 2009; Saragih et al. 2011) which build upon the ASMs. Besides new methodologies, another notable development in the field has been the collection and annotation of large facial data sets captured in unconstrained conditions (in-the-wild) (Belhumeur et al. 2011; Zhu and Ramanan 2012; Le et al. 2012; Sagonas et al. 2013). Being able to capitalize on large amounts of data, a number of (cascaded) regression-based techniques have been recently proposed which achieve impressive performance (Valstar et al. 2010; Cao et al. 2012; Xiong and De la Torre 2013; Sun et al. 2013; Ren et al. 2014; Asthana et al. 2014; Kazemi and Josephine 2014). The approaches described in Xiong and De la Torre (2013), Ren et al. (2014), Asthana et al. (2014), Kazemi and Josephine (2014) and Tzimiropoulos (2015) along with the part-based generative deformable model of Tzimiropoulos and Pantic (2014) are considered to be the state-of-the-art in face alignment. Regarding AAMs, and following Tzimiropoulos and Pantic (2013), there have been a few notable approaches to AAM fitting, see for example Kossaifi et al. (2014) and Kossaifi et al. (2015). State-of-the-art is considered the part-based AAM of Tzimiropoulos and Pantic (2014).

## Active Appearance Models

An AAM is defined by the shape, appearance and motion models. Learning the shape model requires consistently annotating a set of *u* landmarks $[x_1,y_1, \ldots x_u, y_u]$ across *D* training images $\mathbf {I}_i(\mathbf {x})$ (e.g. faces). These points are said to define the shape of each object. Next, Procrustes Analysis is applied to remove similarity transforms from the original shapes and obtain *D* similarity-free shapes. Finally, PCA is applied on these shapes to obtain a shape model defined by the mean shape $\mathbf {s}_{0}$ and *n* shape eigenvectors $\mathbf {s}_i$ compactly represented as columns of matrix $\mathbf {S} \in \mathcal {R}^{2u \times n}$ (note that by construction $\mathbf {S}^T\mathbf {S}=\mathbf {E}$, where $\mathbf {E}$ is the identity matrix). To account for similarity transforms, $\mathbf {S}$ is appended with 4 similarity eigenvectors and re-orthonormalized^1^. An instance of the shape model $\mathbf {s}(\mathbf {p})$ is given by

1 $$\begin{aligned} \mathbf {s}(\mathbf {p})=\mathbf {s}_0 + \mathbf {S} \mathbf {p}, \end{aligned}$$

where $\mathbf {p} \in \mathcal {R}^{n \times 1}$ is the vector of the shape parameters.

Learning the appearance model requires removing shape variation from the texture. This can be achieved by first warping each $\mathbf {I}_i$ to the reference frame defined by the mean shape $\mathbf {s}_0$ using motion model $\mathbf {W}$. Finally, PCA is applied on the shape-free textures, to obtain the appearance model defined by the mean appearance $\mathbf {A}_0$, and *m* appearance eigenvectors $\mathbf {A}_i$ compactly represented as columns of matrix $\mathbf {S} \in \mathcal {R}^{N \times m}$ (similarly, $\mathbf {A}^T\mathbf {A}=\mathbf {E}$). An instance of the appearance model $\mathbf {A}(\mathbf {c})$ is given by

2 $$\begin{aligned} \mathbf {A}(\mathbf {c})=\mathbf {A}_0 + \mathbf {A} \mathbf {c}, \end{aligned}$$

where $\mathbf {c} \in \mathcal {R}^{m\times 1}$ is the vector of the appearance parameters.

We used piecewise affine warps $\mathbf {W}(\mathbf {x};\mathbf {p})$ as the motion model for AAMs in this work. Briefly, to define a piecewise affine warp, one firstly needs to triangulate the set of vertices of the given shapes. Then, each triangle in $\mathbf {s}(\mathbf {p})$ and the corresponding triangle in $\mathbf {s}_0$ are used to define an affine warp. The collection of all affine warps defines a piecewise affine warp which is parameterized with respect to $\mathbf {p}$.

Finally, a model instance is synthesized to represent a test object by warping $\mathbf {A}(\mathbf {c})$ from the mean shape $\mathbf {s}_0$ to $\mathbf {s}(\mathbf {p})$ using the piecewise affine warp $\mathbf {W}(\mathbf {x};\mathbf {p})$ defined by $\mathbf {s}(\mathbf {p})$ and $\mathbf {s}_0$. Please see Cootes et al. (2001) and Matthews and Baker (2004) for a detailed coverage of AAMs.

## Background on Fitting AAMs

Given a test image $\mathbf {I}$, inference in AAMs entails estimating $\mathbf {p}$ and $\mathbf {c}$ assuming reasonable initialization of the fitting process. This initialization is typically performed by placing the mean shape according to the output of an object (in this work face) detector. Note that only $\mathbf {p}$ needs to be estimated for localizing the landmarks. Estimating $\mathbf {c}$ is a by-product of the fitting algorithm. Various algorithms and cost functions have been proposed to estimate $\mathbf {p}$ and $\mathbf {c}$ including regression, classification and non-linear optimization methods. The latter approach is of particular interest in this work. It minimizes the $\ell _2$-norm of the error between the model instance and the given image with respect to the model parameters as follows

3 $$\begin{aligned} \arg \min _{\mathbf {p},\mathbf {c}} ||\mathbf {I}[\mathbf {p}]- \mathbf {A}_0 - \mathbf {A} \mathbf {c}||^2, \end{aligned}$$

where for notational convenience we write $\mathbf {I}[\mathbf {p}](k)$ to denote the pixel intensity $\mathbf {I}(\mathbf {W}(\mathbf {x}_k; \mathbf {p}))$, and $\mathbf {I}[\mathbf {p}]$ to denote image $\mathbf {I}(\mathbf {W}(\mathbf {x}; \mathbf {p}))$ re-arranged as a $N \times 1$ vector.

In a series of seminal papers (Baker et al. 2003; Matthews and Baker 2004), the authors illustrated that problem (3) can be solved using using an optimization framework based on a generalization of the Lucas–Kanade (LK) algorithm (Lucas et al. 1981). In particular, because (3) is a non-linear function of $\mathbf {p}$, the standard approach to proceed is to linearize with respect to the shape parameters $\mathbf {p}$ and then optimize iteratively in a Gauss–Newton fashion. As illustrated in Baker et al. (2003); Matthews and Baker (2004), linearization of (3) with respect to $\mathbf {p}$ can be performed in two coordinate frames. In the *forward* case, the test image $\mathbf {I}$ is linearized around the current estimate $\mathbf {p}$, a solution for a $\varDelta \mathbf {p}$ is sought using least-squares, and $\mathbf {p}$ is updated in an additive fashion $\mathbf {p}\leftarrow \mathbf {p} + \varDelta \mathbf {p}$. In general, forward algorithms are slow because the Jacobian and its inverse must be re-evaluated at each iteration.

Fortunately, computationally efficient algorithms can be derived by solving (3) using the inverse compositional framework. Let $\mathbf {A}_i$ represent the i-th column (basis) of $\mathbf {A}$. In inverse algorithms, each basis $\mathbf {A}_i$ is linearized around $\mathbf {p} = 0$. By additionally linearizing with respect to $\mathbf {c}$, (3) becomes

4 $$\begin{aligned} \arg \min _{\varDelta \mathbf {p}, \varDelta \mathbf {c}} ||\mathbf {I}[\mathbf {p}]-\mathbf {A}_{0} - \mathbf {J}_0 \varDelta \mathbf {p} - \sum _{i=1}^m \left( c_i+\varDelta c_i\right) \left( \mathbf {A}_{i}+\mathbf {J}_i \varDelta \mathbf {p}\right) ||^2, \end{aligned}$$

where $\mathbf {J}_{i}$ is the $N \times n$ matrix each row of which contains the $1\times n$ vector $[\mathbf {A}_{i,x}[\mathbf {p}](k) \;\; \mathbf {A}_{i,y}[\mathbf {p}](k)]\frac{\partial \mathbf {W}(\mathbf {x}_k;\mathbf {p})}{\partial \mathbf {p}}$. $\mathbf {A}_{i,x}[\mathbf {p}](k)$ and $\mathbf {A}_{i,y}[\mathbf {p}](k)$ are the *x* and *y* gradients of $\mathbf {A}_i$ for the $k-$th pixel and $\frac{\partial \mathbf {W}(\mathbf {x}_k;\mathbf {p})}{\partial \mathbf {p}} \in \mathcal {R}^{2 \times n}$ is the Jacobian of the piecewise affine warp. Please see Matthews and Baker (2004) for calculating and implementing $\frac{\partial \mathbf {W}}{\partial \mathbf {p}}$. All these terms are defined in the model coordinate frame for $\mathbf {p}=\mathbf {0}$ and can be pre-computed. An update for $\varDelta \mathbf {c}$ and $\varDelta \mathbf {p}$ can be obtained in closed form only after second order terms are omitted as follows

5 $$\begin{aligned} \arg \min _{\varDelta \mathbf {p}, \varDelta \mathbf {c}} ||\mathbf {I}[\mathbf {p}]-\mathbf {A}_0 - \mathbf {A} \mathbf {c} - \mathbf {A} \varDelta \mathbf {c} - \mathbf {J} \varDelta \mathbf {p}||^2, \end{aligned}$$

where $\mathbf {J} = \mathbf {J}_0 + \sum _{i=1}^{m}c_i\mathbf {J}_i$. In Baker et al. (2003), the update was derived as

6 $$\begin{aligned}{}[\varDelta \mathbf {p}; \varDelta \mathbf {c}] = \mathbf {H}_{sic}^{-1}\mathbf {J}_{sic}^T\big (\mathbf {I}[\mathbf {p}]-\mathbf {A}_0 - \mathbf {A} \mathbf {c}\big ), \end{aligned}$$

where $\mathbf {J}_{sic} = [\mathbf {A}; \mathbf {J}] \in \mathcal {R}^{N \times (m+n)}$ and $\mathbf {H}_{sic} = \mathbf {J}_{sic}^T \mathbf {J}_{sic}$. Once $\varDelta \mathbf {p}$ is computed, $\mathbf {p}$ is updated in a compositional fashion $\mathbf {p}\leftarrow \mathbf {p} \circ \varDelta \mathbf {p}^{-1}$, where $\circ$ denotes the composition of two warps. Please see Matthews and Baker (2004) for a principled way of applying the inverse composition to AAMs. This is the well-known Simultaneous Inverse Compositional (SIC) algorithm. The simultaneous algorithm is very slow because the Jacobian $\mathbf {J}_{sic}$, the Hessian $\mathbf {H}_{sic}$ and its inverse must be re-computed at each iteration. One can easily show that the cost for the Hessian computation and its inverse is $O((m+n)^2N + (m+n)^3)$.

Although SIC is very slow, more efficient ways to optimize exist. In particular, the most efficient algorithm for fitting AAMs is the so-called Project-Out Inverse Compositional (POIC) algorithm, which in essence is a LK algorithm. This algorithm decouples shape and appearance by solving (5) in the subspace orthogonal to $\mathbf {A}$. Let us define the projection operator $\mathbf {P}_A = \mathbf {E}-\mathbf {A}\mathbf {A}^T$, where $\mathbf {E}$ is the identity matrix. Observe that $||\mathbf {I}[\mathbf {p}]-\mathbf {A}_0 - \mathbf {A} \mathbf {c}||_{\mathbf {P}_A}^2 = ||\mathbf {I}[\mathbf {p}]-\mathbf {A}_0 ||_{\mathbf {P}_A}^2$ 2. Hence an update for $\varDelta \mathbf {p}$ can be computed by optimizing

7 $$\begin{aligned} \arg \min _{\varvec{\Delta } \mathbf {p}} ||\mathbf {I}[\mathbf {p}]-\mathbf {A}_0 - \mathbf {J}_0 \varDelta \mathbf {p}||_{\mathbf {P}_A}^2. \end{aligned}$$

The solution to the above problem is given by

8 $$\begin{aligned} \varDelta \mathbf {p} = \mathbf {H}_{poic}^{-1}\mathbf {J}_{poic}^T\big (\mathbf {I}[\mathbf {p}]-\mathbf {A}_0 \big ), \end{aligned}$$

where the projected-out Jacobian $\mathbf {J}_{poic}=\mathbf {P}_A\mathbf {J}_{0}$ and Hessian $\mathbf {H}_{poic} = \mathbf {J}_{poic}^T\mathbf {J}_{poic}$, can be both pre-computed. This reduces the cost per iteration to *O*(*nN*), only (Matthews and Baker 2004), which is the cost of the inverse compositional LK algorithm (Baker et al. 2003). This algorithm has been shown to track faces at 300 fps (Gross et al. 2005).

## An Optimization Framework for Efficient Fitting of AAMs

Solving the exact problem in a simultaneous fashion as described above is not the only way for robust fitting of AAMs. In this section, we derive and solve the optimization problem for Fast-SIC, a fast algorithm that is theoretically guaranteed to provide exactly the same updates per iteration as the ones provided by SIC in $O(nmN + n^2N)$. The derived update rules for Fast-SIC were originally proposed in Hager and Belhumeur (1998) and applied to the problem of AAM fitting in Papandreou and Maragos (2008). In this section, we provide a derivation based on a standard result from optimization theory (Eq. (9)), which has the advantage of producing the exact form of the optimization problem that Fast-SIC solves. Our derivation sheds further light on the different optimization problems that POIC and SIC solve and shows that POIC is only an approximation to Fast-SIC (and hence to SIC). Additionally, we describe a simple and approximate Fast-SIC algorithm which we coin aFast-SIC with similar fitting and convergence performance (as experimentally shown) but with complexity $O((n+m)N+n^2N)$. Both algorithms, and especially aFast-SIC, are not only computationally realizable but also relatively attractive speed-wise for most current systems. Finally, it is worth noting that based on the analysis presented in this section, in the next section, we describe an algorithm which is shown to largely outperform POIC, and similarly to POIC, has complexity *O*(*nN*), only.

Let *f* be a function that is not necessarily convex. Then a standard result from optimization theory is Boyd and Vandenberghe (2004)

9 $$\begin{aligned} \min _{x,y}f(x,y) = \min _x\big [\min _yf(x,y)\big ]. \end{aligned}$$

Using (9), we can optimize (5) with respect to $\varDelta \mathbf {c}$, and then plug in the solution (which will be a function of $\varDelta \mathbf {p}$) back to (5). Then, we can optimize (9) with respect to $\varDelta \mathbf {p}$. Setting the derivative of (5) with respect to $\varDelta \mathbf {c}$ equal to $\mathbf {0}$ gives the update of $\varDelta \mathbf {c}$ (see appendix for a detailed derivation)

10 $$\begin{aligned} \varDelta \mathbf {c}= & {} \big (\mathbf {A}^T\mathbf {A}\big )^{-1}\mathbf {A}^T\big (\mathbf {I}-\mathbf {A}_0 - \mathbf {A} \mathbf {c} - \mathbf {J} \varDelta \mathbf {p}\big )\nonumber \\= & {} \mathbf {A}^T\big (\mathbf {I}-\mathbf {A}_0 - \mathbf {A} \mathbf {c} - \mathbf {J} \varDelta \mathbf {p}\big ). \end{aligned}$$

Plugging the above into (5) we get the following optimization problem

11 $$\begin{aligned} \arg \min _{\varvec{\Delta } \mathbf {p}} ||\mathbf {I}-\mathbf {A}_0 -\mathbf {J} \varDelta \mathbf {p}||_{\mathbf {P}_A}^2, \end{aligned}$$

the solution of which is readily given by

12 $$\begin{aligned} \varDelta \mathbf {p} = \mathbf {H}_{fsic}^{-1}\mathbf {J}_{fsic}^T\big (\mathbf {I}-\mathbf {A}_0\big ), \end{aligned}$$

where the projected-out Jacobian and Hessian are given by $\mathbf {J}_{fsic}=\mathbf {P}_A\mathbf {J}$ and $\mathbf {H}_{fsic} = \mathbf {J}_{fsic}^T\mathbf {J}_{fsic}$, respectively. Because $\mathbf {J}$ is a function of $\mathbf {c}$, it needs to be re-computed per iteration.

As we may see from (11), the difference between POIC and Fast-SIC (and hence SIC) is that POIC uses $\mathbf {J}_0$ while Fast-SIC uses $\mathbf {J}$. This difference simply comes from the point at which we choose to linearize. The authors in Matthews and Baker (2004) chose to project out first and then linearize. Fast-SIC first linearizes the appearance model, and then projects out. Overall, it is evident that because the Jacobian $\mathbf {J}$ has been omitted from (8), POIC and Fast-SIC produce different solutions per iteration. Hence POIC is only an approximation to Fast-SIC (and hence to SIC). We attribute the large performance gap (as we show later on) between Fast-SIC and POIC to this approximation.

Another way to interpret Fast-SIC is to solve the original SIC problem of (5) in the subspace defined by $\mathbf {P}_A$. This has the effect that the appearance terms $\mathbf {A}\mathbf {c}$ and $\mathbf {A}\varDelta \mathbf {c}$ immediately vanish. However, the Jacobian $\mathbf {J}_c = \sum _{i=1}^{m}c_i\mathbf {J}_i$ does not belong to the appearance subspace $\mathbf {A}$, and therefore does not vanish as assumed by POIC. To make it vanish, in the next section, we propose an algorithm which works in the subspace orthogonal to both $\mathbf {A}$ and $\mathbf {J}_i$, and is shown to largely outperform POIC.

To calculate the cost for Fast-SIC, we just note that for a matrix $\mathbf {X}\in \mathcal {R}^{N \times l}$, we can calculate $\mathbf {P}_A\mathbf {X}=\mathbf {X} - \mathbf {A}(\mathbf {A}^T\mathbf {X})$ with cost *O*(*lmN*). Hence, the complexity per iteration is *O*(*nmN*) for computing $\mathbf {J}_{fsic}$, $O(n^2N)$ for computing $\mathbf {H}_{fsic}$ and $O(n^3)$ for inverting $\mathbf {H}_{fsic}$. Because typically $m \gg n$, the main computational burden is $O(nmN+n^2N)$ and is related to the calculation of the projected-out Jacobian $\mathbf {J}_{fsic}$.

The above cost can be readily reduced by using a simple approximation: when computing (12), we can write $\mathbf {J}_{fsic}^T(\mathbf {I}-\mathbf {A}_0)=\mathbf {J}^T\mathbf {P}_A^T(\mathbf {I}-\mathbf {A}_0)$. Now $\mathbf {P}_A^T(\mathbf {I}-\mathbf {A}_0)$ takes *O*(*mN*) and one can compute $\mathbf {J}$ as the Jacobian of $\mathbf {A}(\mathbf {c})$ also in *O*(*mN*). Hence, if we approximate $\mathbf {H}_{fsic}$ with $\mathbf {H} = \mathbf {J}^T\mathbf {J}$, the overall cost of the algorithm is reduced to $O((n+m)N+n^2N)$. We call this algorithm approximate Fast-SIC (aFast-SIC). Note that aFast-SIC can be readily seen as a block coordinate descent algorithm for minimizing (5). In particular, by keeping $\varDelta \mathbf {p}$ fixed and optimizing with respect to $\varDelta \mathbf {c}$, as we showed above, we can readily derive (10). Then, by keeping $\varDelta \mathbf {c}$ fixed and optimizing with respect to $\varDelta \mathbf {p}$, we can readily derive the unprojected Hessian and the same update as the one employed by aFast-SIC. As we show below aFast-SIC achieves essentially the same fitting and convergence performance as the one achieved by Fast-SIC.

## Extended Project-Out Inverse Compositional (E-POIC) Algorithm

Although Fast-SIC and especially aFast-SIC reduce the complexity of the original SIC algorithm dramatically (from $O((n+m)^2N+n^2N)$ to $O((n+m)N+n^2N)$, they still require expensive matrix multiplications per iteration, and significant memory requirements (both the appearance model and its gradients must be stored in memory). To address these limitations, regression approaches to AAM fitting attempt to learn a mapping $\mathbf {K}\in R^{n \times N}$ between the error image $\mathbf {E}_{r}=\mathbf {I}[\mathbf {p}]-\mathbf {A}_0$ and the update of the shape parameters

13 $$\begin{aligned} \varDelta \mathbf {p} = \mathbf {K}\mathbf {E}_r, \end{aligned}$$

where $\mathbf {K}$ is typically estimated via linear regression. Note that the complexity per iteration of the above equation is only *O*(*nN*).

We note that although derived from a totally different pathway, POIC is similar to regression-based approaches having a computational cost of *O*(*nN*). This can be readily seen by writing $\mathbf {K}=\mathbf {H}_{p}^{-1}\mathbf {J}^T$ and $\mathbf {E}_r=\mathbf {P}_A^T(\mathbf {I}[\mathbf {p}]-\mathbf {A}_0)$. Unfortunately, as our experiments hereafter show, there is a very large difference in performance between POIC and Fast-SIC. Based on the analysis presented in Sect. 5, in the following subsections, we describe the Extended Project-Out Inverse Compositional (E-POIC) algorithm, a very fast gradient descent-based project-out algorithm with complexity *O*(*nN*) which largely outperforms POIC and bridges the gap with Fast-SIC. E-POIC is a combination of two algorithms E-POIC-v1 and E-POIC-v2 which both outperform POIC whilst the individual performance improvements turn out to be orthogonal.

### E-POIC-v1: Project-Out the Steepest Descent Images

As noted in Sect. (5), the Jacobian $\mathbf {J}_c = \sum _{i=1}^{m}c_i\mathbf {J}_i$ does not belong to the appearance subspace $\mathbf {A}$, and therefore does not vanish as assumed by POIC. Hence POIC is only an approximation to Fast-SIC. It turns out that this approximation deteriorates performance significantly. To alleviate this problem, we propose to solve (5) in the subspace orthogonal to *both* $\mathbf {A}$ *and* $\mathbf {J}_i, i=1, \dots , m$. More, specifically, let us write $\mathbf {J}_c$ as the concatenation of *n* columns

14 $$\begin{aligned} \mathbf {J}_c = \sum _{i=1}^{m}c_i\mathbf {J}_i = \big [\mathbf {J}^{1}\mathbf {c}\;\ldots \; \mathbf {J}^{n}\mathbf {c}\big ], \end{aligned}$$

where each column $\mathbf {J}^{j}\mathbf {c} = \sum _{i=1}^{m}c_i\mathbf {J}^{j}_{i},\;\; j=1,\ldots ,n$, and $\mathbf {J}^{j}_{i}$ is the j-th column of $\mathbf {J}_{i}$. Following Baker et al. (2003), $\mathbf {J}^{j}_{i},\;\; j=1,\ldots ,n$ are called the steepest descent images of $\mathbf {A}_i$. We now define a subspace for each $\mathbf {J}^{j}$, $\varvec{\Phi }_i \in \mathcal {R}^{N\times l}$, and a concatenation of subspaces $\varvec{\Phi }=[\varvec{\Phi }_1\ldots \varvec{\Phi }_n]$. Finally, we define an extended subspace $\mathbf {A}_{\varPhi } = [\mathbf {A} \; \; \varvec{\Phi }]$ for modelling the appearance variation of both training and steepest descent images and the extended projection operator $\mathbf {P}_{\varPhi }=\mathbf {E}-\mathbf {A}_{\varPhi }(\mathbf {A}_{\varPhi }^T\mathbf {A}_{\varPhi })^{-1}\mathbf {A}_{\varPhi }^T$. Notice that if all components corresponding to non-zero eigenvalues in $\mathbf {P}_{\varPhi }$ are preserved, we can write

15 $$\begin{aligned} ||\mathbf {I}[\mathbf {p}]-\mathbf {A}_0 - \mathbf {A} \mathbf {c} - \mathbf {A} \varDelta \mathbf {c} - \mathbf {J} \varDelta \mathbf {p})||_{\mathbf {P}_{\varPhi }}^2 = ||\mathbf {I}[\mathbf {p}]-\mathbf {A}_0 - \mathbf {J}_0 \varDelta \mathbf {p} ||_{\mathbf {P}_{\varPhi }}^2. \end{aligned}$$

The proposed optimization problem is therefore given by

16 $$\begin{aligned} \arg \min _{\varvec{\Delta } \mathbf {p}} ||\mathbf {I}[\mathbf {p}]-\mathbf {A}_0 - \mathbf {J}_0 \varDelta \mathbf {p}||_{\mathbf {P}_{\varPhi }}^2, \end{aligned}$$

the solution of which is given by (8) where $\mathbf {P}_A$ is replaced by $\mathbf {P}_{\varPhi }$ and hence the cost per iteration is *O*(*nN*). We coin this algorithm E-POIC-v1.

### E-POIC-v2: Project-Out Joint Alignment

POIC algorithm essentially averages over all training images and performs alignment between the mean appearance and the test image. As we show below, this introduces some undesirable terms in the calculation of the Hessian which deteriorate fitting performance. To alleviate this, we propose to jointly align the test image with all training images and then average out the result in a similar fashion to regression approaches. This can be also seen as one iteration of the so-called joint alignment framework of “image congealing” (Huang et al. 2007; Cox et al. 2008). Because all training images are already aligned to each other, we show below that this idea can be extended within the project-out inverse compositional framework with complexity *O*(*nN*).

More specifically, suppose that we perform a Taylor expansion of each training image $\mathbf {I}_i$ at $\mathbf {p}=\mathbf {0}$, $\mathbf {I}_i = \mathbf {I}_i[\mathbf {0}] + \mathbf {G}_i\varDelta \mathbf {p}$, where $\mathbf {G}_i\in \mathcal {R}^{N \times n}$ is the Jacobian of image $\mathbf {I}_i$ evaluated at $\mathbf {p}=\mathbf {0}$. We propose to compute an update for $\varDelta \mathbf {p}$ by solving the following problem

17 $$\begin{aligned} \arg \min _{\varvec{\Delta } \mathbf {p}} \sum _i ||\mathbf {I}[\mathbf {p}]-\mathbf {I}_i - \mathbf {G}_i \varDelta \mathbf {p}||^2. \end{aligned}$$

Due to appearance variation, there is a mismatch between $\mathbf {I}[\mathbf {p}]$ and $\mathbf {I}_i$. To compensate for this, we further propose to solve (17) in a subspace which removes appearance variation. Suppose that $\mathbf {I}_i = \mathbf {A}_0 + \mathbf {A}\mathbf {c}_i$. Then, we propose to solve the following optimization problem

18 $$\begin{aligned} \arg \min _{\varvec{\Delta } \mathbf {p}} \sum _i ||\mathbf {I}[\mathbf {p}]-\mathbf {A}_0 - \mathbf {A}\mathbf {c}_i - \mathbf {G}_i \varDelta \mathbf {p}||_{\mathbf {P}_A}^2. \end{aligned}$$

The solution to the above problem is readily given by

19 $$\begin{aligned} \varDelta \mathbf {p} = \mathbf {H}_{ja}^{-1}\mathbf {J}_{ja}^T\big (\mathbf {I}[\mathbf {p}]-\mathbf {A}_0\big ), \end{aligned}$$

where $\mathbf {J}_{ja} = \mathbf {P}_A\sum _i\mathbf {G}_i = \mathbf {P}_A\mathbf {J}_0$ and $\mathbf {H}_{ja}=\sum _i\mathbf {G}_i^T\mathbf {P}_A\mathbf {G}_i$ can be both pre-computed. Hence, the cost per iteration is *O*(*nN*). We coin this algorithm E-POIC-v2.

It is worth noting that the difference between POIC and E-POIC-v2 boils down to how the Hessian is calculated. E-POIC-v2 uses an average projected-out Hessian. In contrast, POIC first averages over all images, and then computes the projected-out Hessian of the mean appearance $\mathbf {H}_{poic} = (\sum _i\mathbf {G}_i)^T\mathbf {P}_A\sum _j\mathbf {G}_j$. The result is that $\mathbf {H}_{poic}$ contains cross-terms of the form $\mathbf {G}_i^T\mathbf {P}_A\mathbf {G}_j, i\ne j$ which do not appear in $\mathbf {H}_{ja}$. As our results have shown, these terms deteriorate performance significantly.

### The Extended Project-Out Inverse Compositional Algorithm

We coin the combination of E-POIC-v1 and E-POIC-v2 as the Extended Project-Out Inverse Compositional (E-POIC) algorithm. This algorithm simply replaces $\mathbf {P}_A$ with $\mathbf {P}_{\varPhi }$ in (18). Hence, the proposed optimization problem is

20 $$\begin{aligned} \arg \min _{\varvec{\Delta } \mathbf {p}} \sum _i ||\mathbf {I}[\mathbf {p}]-\mathbf {A}_0 - \mathbf {A}\mathbf {c}_i - \mathbf {G}_i \varDelta \mathbf {p}||_{\mathbf {P}_\varPhi }^2, \end{aligned}$$

the solution of which is given by (19) where $\mathbf {P}_A$ is replaced by $\mathbf {P}_{\varPhi }$ and hence the cost per iteration is *O*(*nN*).

## Fitting AAMs to Unconstrained Images

In general, fitting AAMs to unconstrained images is considered a difficult task. Perhaps, the most widely acknowledged reason for this is the limited representational power of the appearance model which is unable to generalize well to unseen variations. In particular, all optimization problems considered in the previous sections are least-squares problems, and, as it is well-known in computer vision, least-squares combined with pixel intensities as features typically results in poor performance for data corrupted by outliers (e.g. sunglasses, occlusions, difficult illumination). Standard ways of dealing with outliers are robust norms and robust features. The problem with robust norms is that scale parameters must be estimated (or percentage of outlier pixels must be predefined) and this task is not trivial. The problem with feature extraction is that it might slow down the speed of the fitting algorithm significantly especially when the dimensionality of the featured-based appearance model is large.

**Fig. 1:**
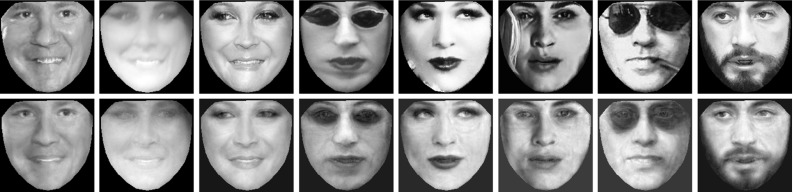
*First row:* face images taken from the test set of LFPW (Belhumeur et al. 2011). The images were not seen during training. *Second row:* reconstruction of the images from the appearance subspace. The appearance subspace is powerful because the AAM was built in the wild

In this section, we propose a third orthogonal direction for employing AAMs in unconstrained conditions by means of training AAMs in-the-wild, and fitting using the proposed fast and robust algorithms (in particular, Fast-SIC, aFast-SIC and E-POIC). Interestingly, the combination of generative models plus training in-the-wild (plus robust optimization for model fitting) has not been thoroughly investigated in literature. It turns out that this combination is very beneficial for unconstrained AAM fitting. Consider for example the images shown in the first row of Fig. 1. These are test images from the LFPW data set. The images were not seen during training, but similar images of unconstrained nature were used to train the shape and appearance model of the AAM. The second row of Fig. 1 shows the reconstruction of the images from the appearance subspace. As we may see, the appearance model is powerful enough to reconstruct the texture almost perfectly. Because reconstruction is feasible, fitting an AAM to these images is also feasible if a robust algorithm is used for model fitting.

To illustrate the boost in robustness obtained by training AAMs in-the-wild, we used the training set of LFPW to train the shape and the appearance model of *pixel-based* AAMs, and the test set of the same database to quantify fitting accuracy. The database consists of images from the web containing large variations in pose, illumination, expression and occlusion. For our experiments, in order to assess performance, we used the same average (computed over all 68 points) point-to-point Euclidean error normalized by the face size as the one used in Zhu and Ramanan (2012). Similarly to Zhu and Ramanan (2012), for this error measure, we produced the cumulative curve corresponding to the percentage of test images for which the error was less than a specific value. In all cases, fitting was initialized by the bounding box of Zhu and Ramanan (2012).

**Fig. 2:**
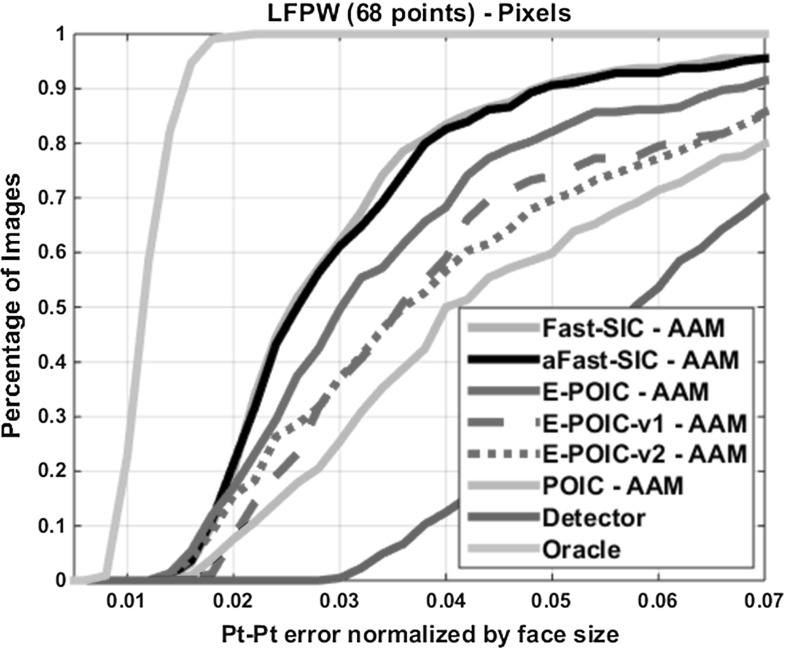
Fitting performance of *pixel-based* AAMs on LPFW. Average pt-pt Euclidean error (normalized by the face size) versus fraction of images

**Fig. 3:**
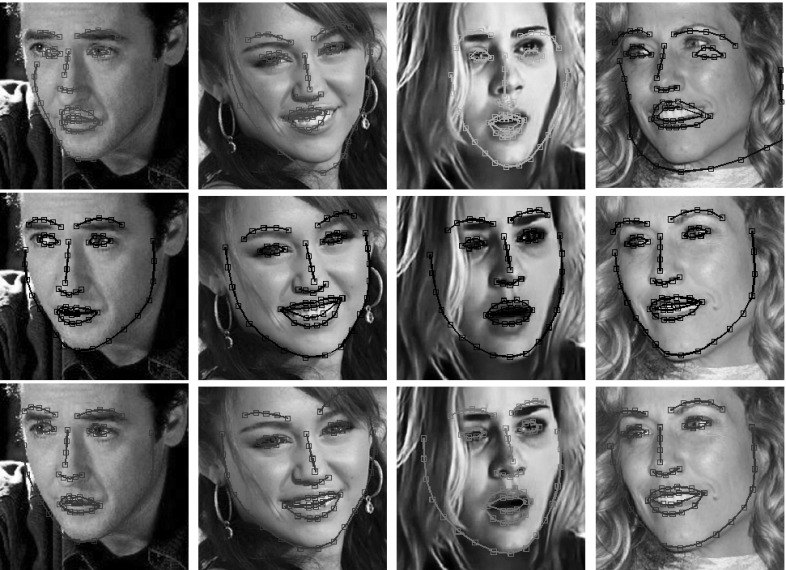
Fitting examples of *pixel-based* AAMs from the test set of LFPW. *First row:* POIC. *Second row:* aFast-SIC. *Third row:* E-POIC. POIC does not perform well, however aFast-SIC and E-POIC achieved satisfactory robustness and accuracy in landmark localization. Notably, to obtain these results, the appearance model of the AAMs was built using raw un-normalized pixel intensities as features. Neither sophisticated shape priors or robust norms were used during fitting nor robust image features were employed to build the AAMs: we simply trained the AAMs in-the-wild on the same database

Figure 2 shows the fitting performance of simultaneous algorithms, namely Fast-SIC and aFast-SIC as well approximate project-out algorithms, namely E-POIC and POIC. Figure 3 shows some fitting examples. As we may observe, Fast-SIC and aFast-SIC feature almost identical performance. As we can additionally observe from Fig. 2, there is a large gap in performance between simultaneous algorithms (Fast-SIC and aFast-SIC) and POIC. However, this gap in performance is largely bridged by the proposed E-POIC which has the same complexity as POIC. In particular, we may observe that E-POIC-v1 performs comparably to E-POIC-v2, and they both outperform POIC by 10–20 % in fitting accuracy. Fortunately, the performance improvements achieved by E-POIC-v1 and E-POIC-v2 turn out to be orthogonal. As we may observe, the overall improvement achieved by E-POIC is almost equal to the summation of the performance improvements achieved by E-POIC-v1 and E-POIC-v2. As we additionally show in Sect. 10, the performance gap between simultaneous algorithms and E-POIC is further reduced when one uses SIFT features to build the appearance model of the AAM.

## Part-Based Active Appearance Models

In our formulation, a part-based AAM is an AAM that draws advantages from the part-based representation and the translational motion model of the Deformable Part Model (DPM) (Felzenszwalb et al. 2010) and (Zhu and Ramanan 2012) (as opposed to the holistic representation and the piecewise affine warp used in standard AAMs). Following Tzimiropoulos and Pantic (2014), we call this model a generative DPM. As we show hereafter, fitting a generative DPM using Gauss–Newton optimization implies a translational motion model which results in more accurate and robust performance compared to that obtained by fitting a standard holistic AAM with the same algorithm. We attribute this performance improvement to the more flexible part-based representation which models only the most relevant parts of the face, and the simplicity of the translational motion model.

### Generative DPM

A generative DPM is described by generative models of global shape and local appearance both learned via PCA, as in the original CLM paper of Cristinacce and Cootes (2008). Unlike Cristinacce and Cootes (2008), both models are kept independent (Matthews and Baker 2004) i.e., we do not apply a third PCA on the embeddings of the shape and texture. The global shape model of the generative DPM is the same as the one used in AAMs, i.e. an instance of the shape model $$\mathbf {s}(\mathbf {p})$$ is given by (1). A key feature of the appearance model is that it is learned from all parts jointly, and hence parts, although capture local appearance, are not assumed independent. The appearance model of the generative DPM is obtained by (a) warping each training image $$\mathbf {I}_i$$ to a reference frame so that similarity transformations are removed, (b) extracting a $$N_p=N_s \times N_s$$ pixel-based part (i.e. patch) around each landmark, (c) obtaining a part-based texture for the whole image by concatenating all parts in a $$N = uN_p $$ vector, and (d) applying PCA to the part-based textures of all training images. In this way, and similarly to an AAM, we obtain the mean appearance $$\mathbf {A}_0$$ and *m* appearance eigenvectors $$\mathbf {A}_i$$, compactly represented as columns of $$\mathbf {A} \in \mathcal {R}^{N \times m}$$. Again, and similarly to an AAM, an instance of the appearance model $$\mathbf {A}(\mathbf {c})$$ is given by (2).

It is worth noting that each $\mathbf {A}_i$ (this also applies to the part-based texture representation of each training image $\mathbf {I}_i$) can be re-arranged as a $u \times N_p$ representation $[\mathbf {A}^{i,1} \; \mathbf {A}^{i,2} \; \ldots \; \mathbf {A}^{i,N_p}]$. Each column $\mathbf {A}^{i,j}\in \mathcal {R}^u$ contains *u* pixels all belonging to a different part but all sharing the same index location *j* within their part. This representation allows us to interpret each patch as a $N_p$-dimensional descriptor for the corresponding landmark. Finally, we define $\mathbf {A}^{j} = [\mathbf {A}^{1,j} \; \mathbf {A}^{2,j} \; \ldots \; \mathbf {A}^{m,j}] \in \mathcal {R}^{u \times m}$.

### Fitting Generative DPMs with Gauss–Newton

Similarly to an AAM, we can fit the generative DPM to a test image using non-linear least-squares optimization. We start by describing the fitting process of a simplified version of the generative DPM by assuming that the patch for each landmark $\mathbf {s}_k$ is reduced to $1\times 1$ ($N_s=1$), that is 1 pixel is used to represent the appearance of each landmark and similarly the appearance model in (2) has a total of $N=u$ pixels. In this case, the construction of the appearance model, in the previous section, implicitly assumes a translational motion model in which each training image is sampled at $N=u$ locations $\mathbf {I}_i(\mathbf {l}_i)$ and then *u* pixels are shifted to a common reference frame which is defined as the frame of the mean shape $\mathbf {s}_0$. In this model, a model instance $\mathbf {M}_y$ is created by first generating *u* pixels using (2) for some $\mathbf {c}=\mathbf {c}_y$ and then shifting these pixels to *u* pixel locations obtained from (1) for some $\mathbf {p}=\mathbf {p}_y$. Hence, we can write

21 $$\begin{aligned} \mathbf {M}_y\big (\mathbf {s}(\mathbf {p}_y)\big ) = \mathbf {A}(\mathbf {c}_y). \end{aligned}$$

The above model can be readily used to locate the landmarks in an unseen image $\mathbf {I}$ using non-linear least-squares. In particular, we wish to find $\{\mathbf {p}, \mathbf {c}\}$ such that

22 $$\begin{aligned} \arg \min _{\mathbf {p},\mathbf {c}} ||\mathbf {I}\big (\mathbf {s}(\mathbf {p})\big )- \mathbf {A}(\mathbf {c})||^2. \end{aligned}$$

Similarly to an AAM, the difference term in the above cost function is linear in $\mathbf {c}$ but non-linear in $\mathbf {p}$. We therefore proceed as in Sect. 5 and derive the same optimization problem as in (5) which, for convenience, we re-write here

23 $$\begin{aligned} \arg \min _{\varDelta \mathbf {p}, \varDelta \mathbf {c}} ||\mathbf {I}-\mathbf {A}(\mathbf {c}) - \mathbf {A} \varDelta \mathbf {c} - \mathbf {J} \varDelta \mathbf {p}||^2, \end{aligned}$$

where, for the case of the generative DPM, $\mathbf {I} = \mathbf {I}\big (\mathbf {s}(\mathbf {p})\big )$, $\mathbf {A}_i = \mathbf {A}_i(\mathbf {s}(\mathbf {p}=\mathbf {0}))= \mathbf {A}_i(\mathbf {s}_0)$, and $\mathbf {J}_i\in \mathcal {R}^{N\times n}$ is the Jacobian of $\mathbf {A}_i$ (notice that $N=u$).

For the translational motion model defined above, we construct $\mathbf {J}_i$ as follows: The $k-$th row of $\mathbf {J}_i$ contains the $1\times n$ vector $[\mathbf {A}_{i,x}(\mathbf {s}_{0,k})\;\;\mathbf {A}_{i,y}(\mathbf {s}_{0,k})]\frac{\partial \mathbf {s}_{k}(\mathbf {p})}{\partial \mathbf {p}}\mid _{\mathbf {p}=\mathbf {0}}$. $\mathbf {A}_{i,x}$ and $\mathbf {A}_{i,y}$ are the *x* and *y* gradients of $\mathbf {A}_i$. To calculate $\frac{\partial \mathbf {s}_k(\mathbf {p})}{\partial \mathbf {p}}\mid _{\mathbf {p}=\mathbf {0}}$, let us also denote by $\mathbf {s}_k=[x_k \; ; \; y_k]$ and $\mathbf {s}_{i,k}=[x_k^{\mathbf {s}_i} \; ; \; y_k^{\mathbf {s}_i}]$ the $k-$th landmark of $\mathbf {s}(\mathbf {p})$ and $\mathbf {s}_i$, respectively. These are related by

24 $$\begin{aligned} \mathbf {s}_k = \big [x_k \; ; \; y_k\big ] = \Bigg [x_k^{\mathbf {s}_0} + \sum _{i=1}^n x_k^{\mathbf {s}_i}\mathbf {p}_i \; ; \; y_k^{\mathbf {s}_0} + \sum _{i=1}^n y_k^{\mathbf {s}_i}\mathbf {p}_i\Bigg ]. \end{aligned}$$

Finally, from (24), we have

25 $$\begin{aligned} \frac{\partial \mathbf {s}_k(\mathbf {p})}{\partial \mathbf {p}}\mid _{\mathbf {p}=\mathbf {0}} = \Big [x_k^{\mathbf {s}_1}\ldots x_k^{\mathbf {s}_n} \; ; \; y_k^{\mathbf {s}_1}\ldots y_k^{\mathbf {s}_n}\Big ] \in \mathcal {R}^{2 \times n}. \end{aligned}$$

To optimize (23), one can use any of the algorithms of Sects. 5 and 6. An interesting deviation from AAMs though is that for the case of our translational motion model inverse composition is reduced to addition. To readily see this, let us first write $\mathbf {s}_{y} = f(\mathbf {s}_{x};\mathbf {p}_a) = \mathbf {s}_{x} + \mathbf {S}\mathbf {p}_a$. Then, $\mathbf {s}_{z} = f(\mathbf {s}_{y};\mathbf {p}_b) = \mathbf {s}_{y} + \mathbf {S}\mathbf {p}_b=\mathbf {s}_{x} + \mathbf {S}\mathbf {p}_a + \mathbf {S}\mathbf {p}_b=\mathbf {s}_{x} + \mathbf {S}(\mathbf {p}_a+\mathbf {p}_b)$, hence composition is reduced to addition. Similarly, we have $f(\mathbf {s}_{x};\mathbf {p}_a)^{-1} = f(\mathbf {s}_{x};-\mathbf {p}_a)$. Overall, inverse composition is reduced to addition, and hence $\mathbf {p}$ can be readily updated in an additive fashion from $\mathbf {p}\leftarrow \mathbf {p}-\varDelta \mathbf {p}$.

Finally, having defined the 1-pixel version of our model, we can now readily move on to the case where the appearance of a landmark is represented by an $N_p = N_s \times N_s$ patch (descriptor) each pixel (element) of which can be seen as a 1-pixel appearance model for the corresponding landmark. Using the $\mathbf {A}^j$ representation defined in Sect. 8.1, the cost function to optimize for GN-DPMs is given by

26 $$\begin{aligned} \arg \min _{\varDelta \mathbf {p}, \varDelta \mathbf {c}} \sum _{j=1}^{N_p} ||\mathbf {I}^j-\mathbf {A}^j(\mathbf {c}) - \mathbf {A}^j \varDelta \mathbf {c} - \mathbf {J}^j \varDelta \mathbf {p}||^2. \end{aligned}$$

By re-arranging the terms above appropriately, it is not difficult to re-write (26) as in (23) where now the error term $\mathbf {I}-\mathbf {A}(\mathbf {c})$ has size $N = uN_p$, and $\mathbf {J}$ has size $N\times n$.

**Fig. 4:**
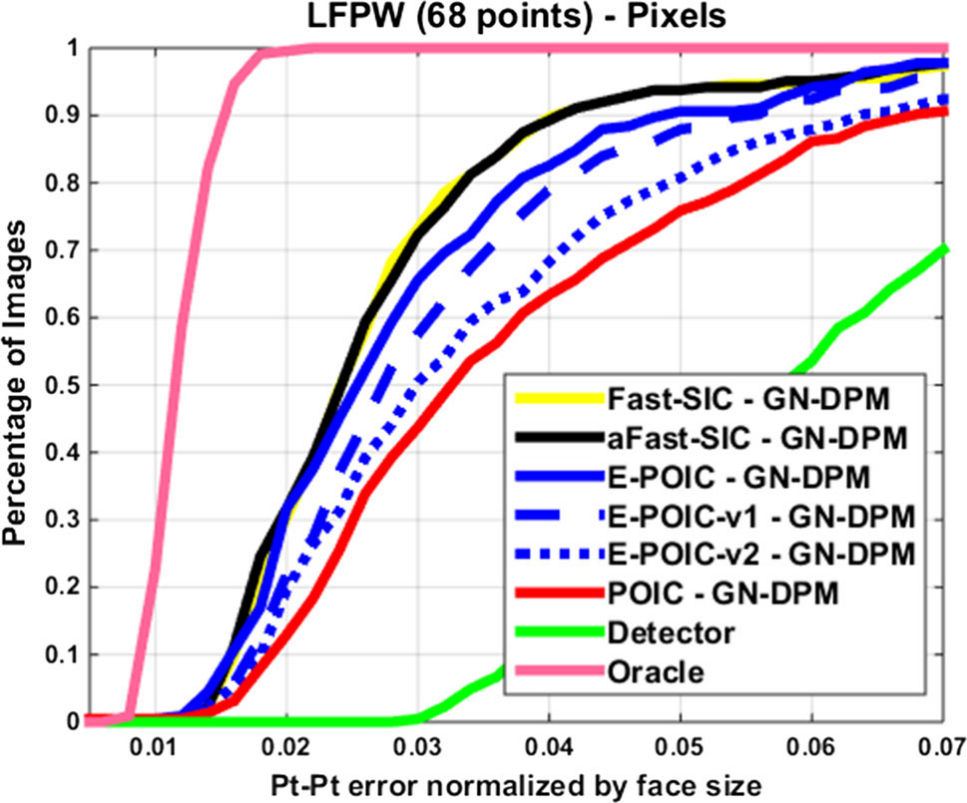
Fitting performance of GN-DPMs on LPFW: Average pt-pt Euclidean error (normalized by the face size) versus fraction of images

We repeated the experiment of Sect. 7 using the same number of shape and appearance parameters for the generative DPM in order to evaluate all algorithms of Sects. 5 and 6. The obtained results are shown in Fig. 4. By comparing these results to those of Fig. 2, we may observe that there is a gain of 10–20 % in fitting accuracy over AAMs. However, the relative difference in performance between all algorithms is similar. As we show later on, the performance gap between simultaneous algorithms and E-POIC is almost negligible when one uses SIFT features to build the appearance model of the generative DPM.

**Fig. 5:**
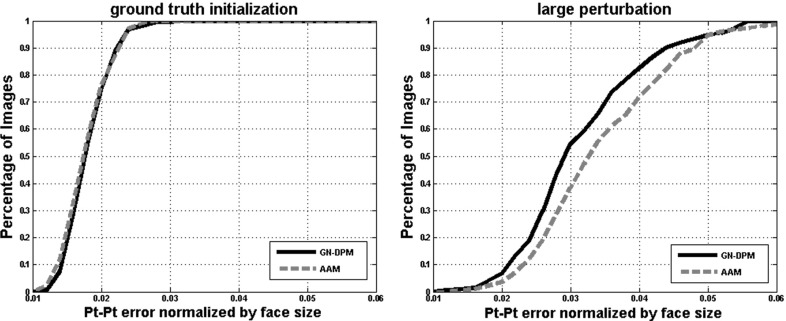
Comparison between GN-DPMs and AAMs. Both algorithms were initialized using the ground truth landmark locations (*left*) and the ground truth after a relatively large perturbation of the first shape parameter (*right*). The average (normalized) pt-pt Euclidean error versus fraction of images is plotted. Clearly GN-DPMs are easier to optimize

**Fig. 6:**
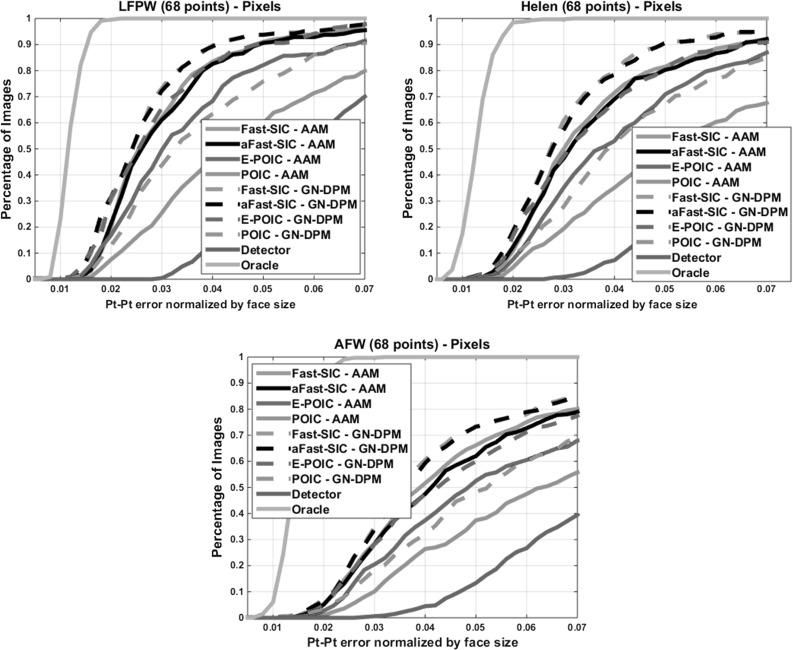
Fitting performance of *pixel-based* AAMs and GN-DPMs on LFPW, Helen and AFW: Average pt-pt Euclidean error (normalized by the face size) versus fraction of images. *68 points* were used

### Comparison with AAMs

Two questions that naturally arise when comparing the part-based GN-DPMs over holistic AAMs are: (a) do both models have the same representational power? and (b) which model is easier to optimize? Because it is difficult to meaningfully compare the representational power of the models directly, in this section, we provide an attempt to shed some light on both questions by conducting an indirect comparison between the two models.

To investigate question (a), we repeated the experiment of Sect. 7 for both GN-DPMs and holistic AAMs, but we initialized both algorithms (we used Fast-SIC for both cases) using the *ground truth* locations of the landmarks for each image. We assume that the more powerful the appearance model is, the better it will reconstruct the appearance of an unseen image, and hence the fitting process will not cause much drifting from the ground truth locations. Fig. 5 (left) shows the obtained cumulative curves for GN-DPMs and AAMs. We may see that both methods achieve literally the same fitting accuracy illustrating that the part-based and holistic approaches have the same representational power. An interesting observation is that the drift from ground truth is very small and the achieved fitting accuracy is very high. This shows that generative deformable models when trained in-the-wild are able to produce a very high degree of fitting accuracy.

To investigate question (b), we reconstructed the ground truth points from the shape model, perturbed the first shape parameter by some amount and then performed fitting using both algorithms. Fig. 5 (right) shows the cumulative curve obtained by applying a relatively large amount of perturbation. Clearly, GN-DPMs largely outperform AAMs. This shows that the part-based generative appearance model of GN-DPMs is easier to optimize.

**Fig. 7:**
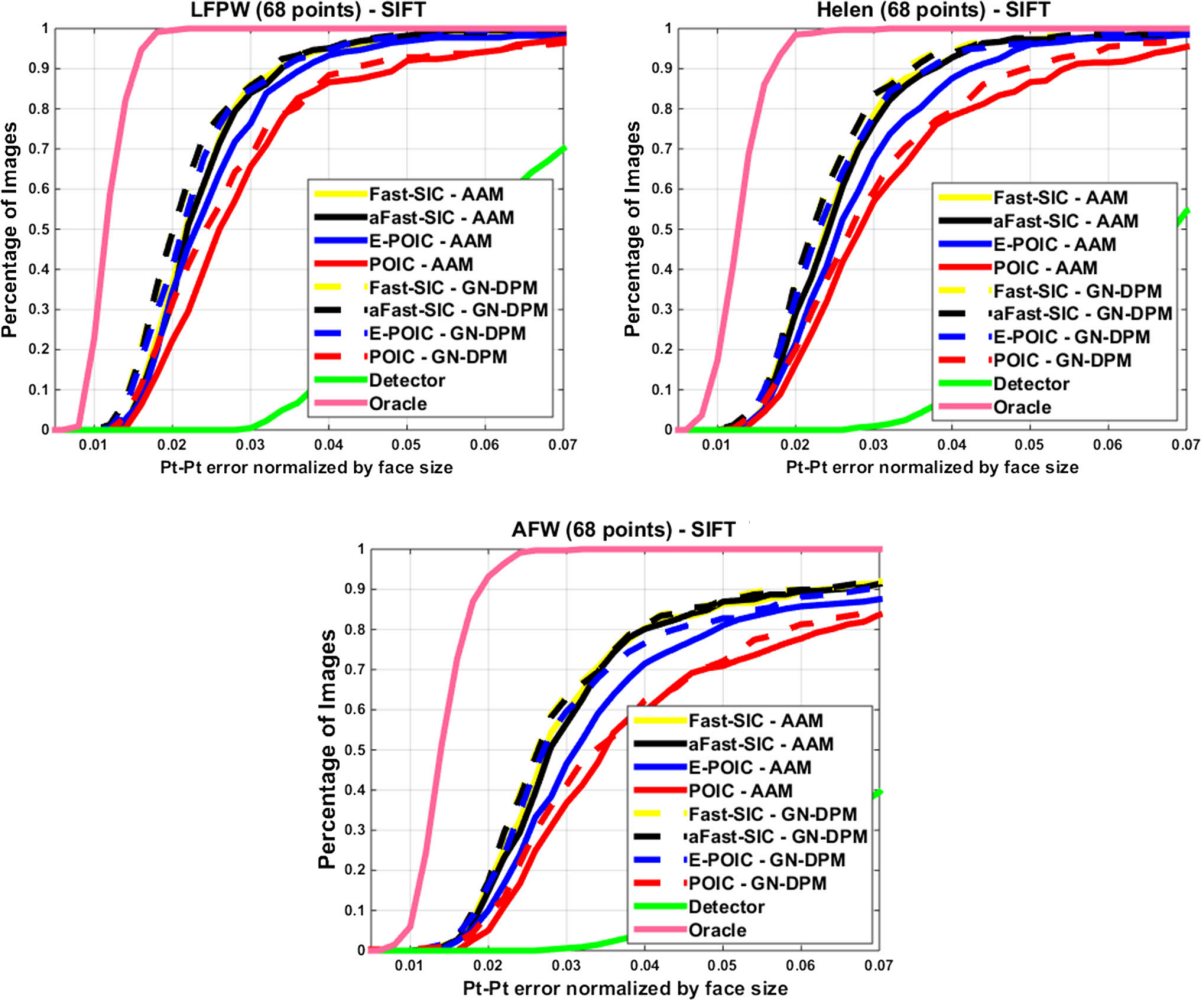
Fitting performance of *SIFT-based* AAMs and GN-DPMs on LFPW, Helen and AFW: Average pt-pt Euclidean error (normalized by the face size) Vs fraction of images. *68 points* were used

**Fig. 8:**
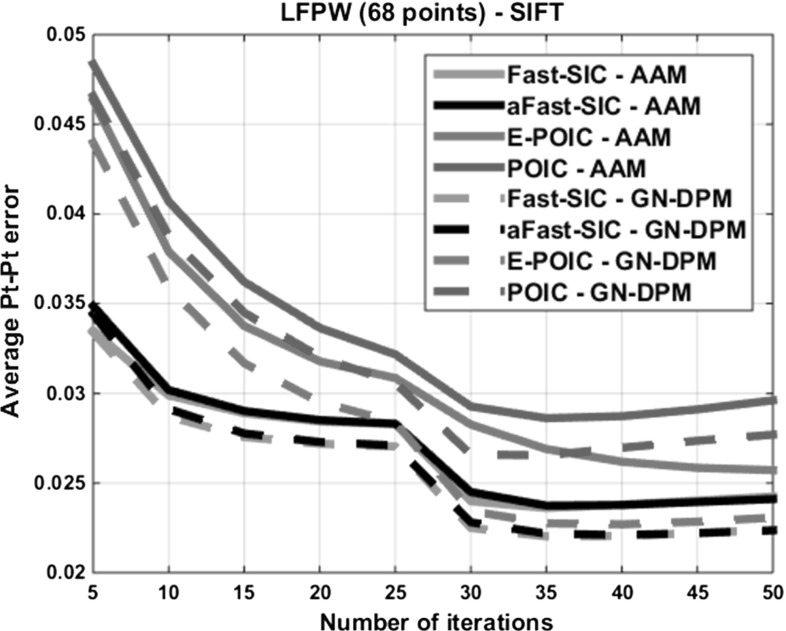
Convergence performance of *SIFT-based* AAMs and GN-DPMs on LPFW: Average pt-pt error versus iteration number

**Fig. 9:**
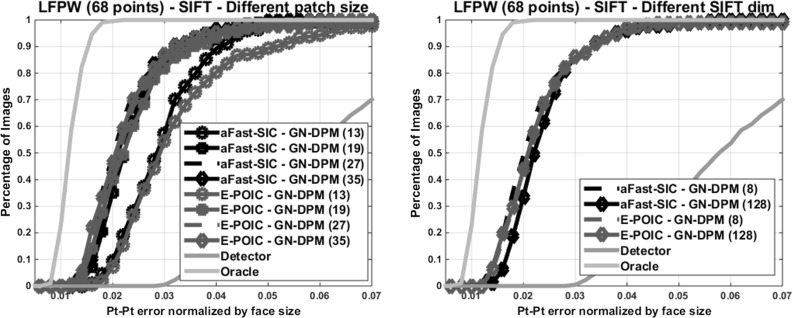
Performance of GN-DPMs for different patch and SIFT sizes. *68 points* were used

**Fig. 10:**
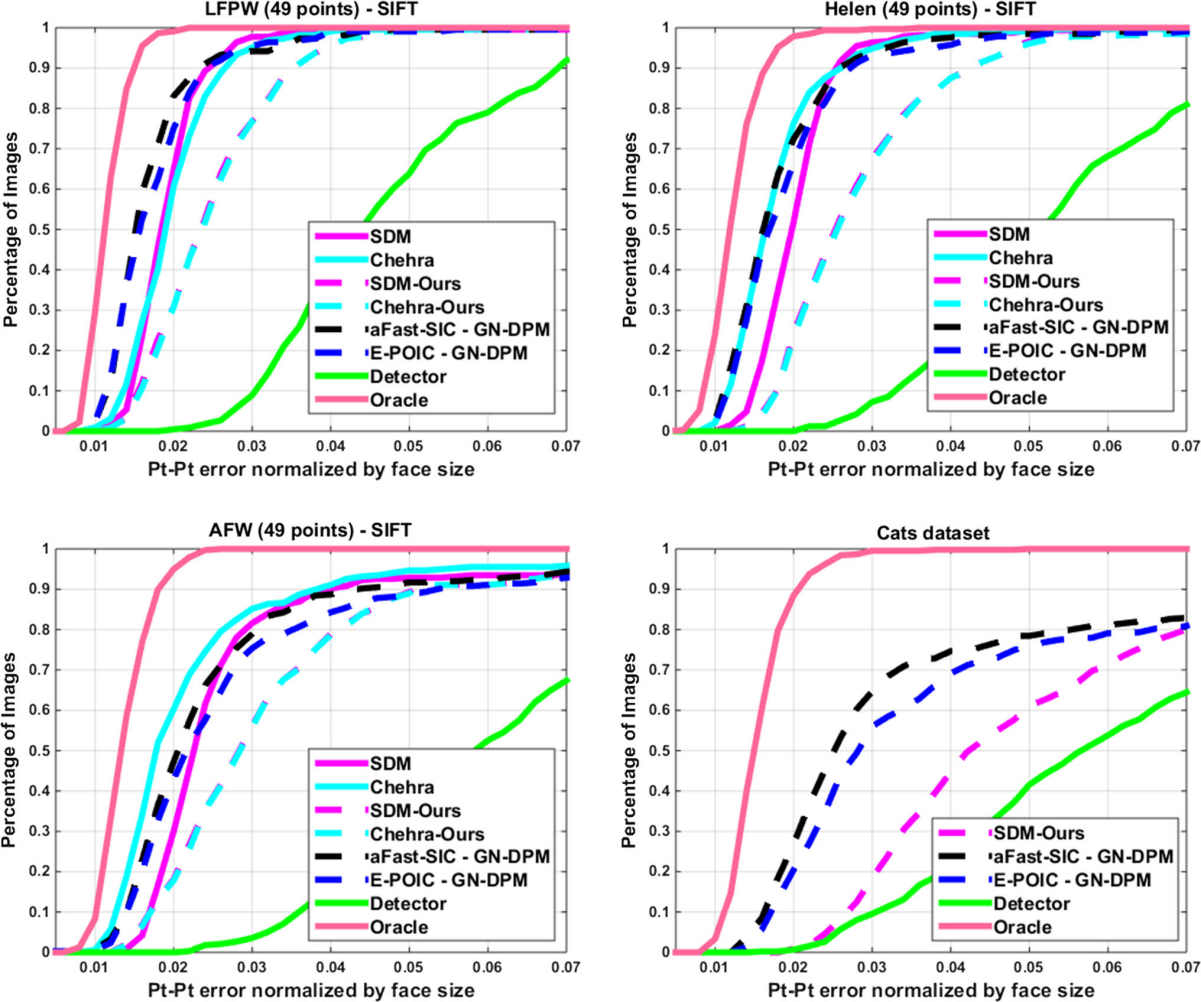
Comparison between *SIFT-AAMs* and SDM and Chehra on LFPW, Helen, AFW and our Cats data set: Average pt-pt Euclidean error (normalized by the face size) versus fraction of images. *49 points* were used for LFPW, Helen and AFW and *42 points* were used for the Cats data set

**Fig. 11:**
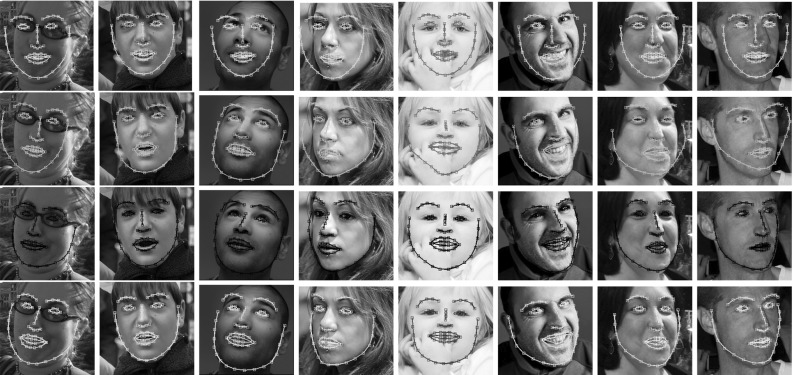
Fitting examples of *SIFT-AAMs* from Helen. *First row:* Detector. *Second row:* POIC-SIFT. *Third row:* aFast-SIC-SIFT. *Fourth row:* E-POIC-SIFT. aFast-SIC-SIFT and E-POIC-SIFT are significantly more robust and accurate than POIC-SIFT

## Efficient Weighted Least-Squares Optimization of SIFT-AAMs

All algorithms presented so far operate on raw pixel intensities. However, one could use other more sophisticated features to boost up robustness and accuracy. In this work, we used the same SIFT features as Xiong and De la Torre (2013) which we found that they produce a large basin of attraction for gradient descent optimization. Building an AAM using SIFT features is straightforward. For example, for the case of standard holistic AAMs (for the case of GN-DPMs, the process is very similar) at each pixel location we extract a SIFT descriptor of dimension $N_f$, and the appearance of each image is represented as a SIFT image (Liu et al. 2008) with $N_f$ channels $\mathbf {I}^k, k=1, \ldots , N_f$. The appearance model of the SIFT-AAM can be learned by warping each $\mathbf {I}^k$ to the mean shape, concatenating all features in a single vector and then applying PCA. In a similar fashion, the mean appearance $\mathbf {A}_0$ and each appearance basis $\mathbf {A}_i$ can be rearranged in $N_f$ channels $\mathbf {A}^k_i, i=1, \ldots , N_f$. Finally for each appearance basis and channel, we can calculate the Jacobian $\mathbf {J}^k_i$ as described in Sect. 4.

Having defined the above notation, both holistic and part-based AAMs, and all algorithms presented so far can be readily extended for the case of SIFT-AAMs. For example, fitting a SIFT-AAM using the Fast-SIC algorithm entails solving the following optimization problem

27 $$\begin{aligned} \arg \min _{\varDelta \mathbf {p}, \varDelta \mathbf {c}} \sum _{k=1}^{N_f} ||\mathbf {I}^{k}[\mathbf {p}] - \mathbf {A}_0^{k}- \mathbf {A}^{k} \varDelta \mathbf {c} - \mathbf {J}^{k} \varDelta \mathbf {p}||_{\mathbf {P}_A}^2. \end{aligned}$$

Using robust features for building the appearance model of an AAM typically increases the complexity of the training, but more importantly, of the fitting process. If the descriptor has length $N_f$, then the size of the appearance model is $N_fN$, and hence complexity increases by a factor of $N_f$. While we used a reduced SIFT representation with $N_f=8$ channels, all resulting fitting algorithms are significantly slower (by a factor of 8) compared to their counterparts built from pixel intensities.

To compensate for this additional computational burden, we propose a fitting approach in which (3) is optimized over a sparse grid of points rather than all points belonging to the convex hull of the mean shape. In particular, this sparse grid is defined by a $N \times N$ diagonal matrix $\mathbf {W}$ the elements of which are equal to 1 corresponding to the locations that we wish to evaluate our cost function and 0 otherwise. Using $\mathbf {W}$, we propose to formulate weighted least-squares problems for all algorithms proposed in this work. In particular, we write

28 $$\begin{aligned} \arg \min _{\varDelta \mathbf {p}, \varDelta \mathbf {c}} ||\mathbf {I}-\mathbf {A}_0-\mathbf {A}\mathbf {c} - \mathbf {A} \varDelta \mathbf {c} - \mathbf {J} \varDelta \mathbf {p})||^2_{\mathbf {W}}. \end{aligned}$$

The question of interest now is whether one can come up with closed-form solutions for $\varDelta \mathbf {c}$ and $\varDelta \mathbf {p}$. Fortunately, the answer is positive. Let us define matrices $\mathbf {A}_w = \mathbf {W}\mathbf {A}$, $\mathbf {J}_{i,w} = \mathbf {W}\mathbf {J}_i$, $\mathbf {J}_w = \mathbf {J}_{0,w} + \sum _{i=1}^{m}c_i\mathbf {J}_{i,w}$, $\mathbf {P}_w=\mathbf {W}-\mathbf {A}_w(\mathbf {A}_w^T\mathbf {A}_w)^{-1}\mathbf {A}_w^T$. Then, for Fast-SIC (similar update rules can be derived for all other algorithms described in this paper) we can update $\varDelta \mathbf {c}$ and $\varDelta \mathbf {p}$ in alternating fashion from

29 $$\begin{aligned} \varDelta \mathbf {c}= & {} \big (\mathbf {A}_w^T\mathbf {A}_w\big )^{-1}\mathbf {A}_w^T\Big (\mathbf {W}\big (\mathbf {I}- \mathbf {A} (\mathbf {c})\big ) - \mathbf {J}_w \varDelta \mathbf {p}\Big ) \end{aligned}$$

30 $$\begin{aligned} \varDelta \mathbf {p}= & {} \mathbf {H}_{wfsic}^{-1}\mathbf {J}_{wfsic}^T\Big (\mathbf {W}\big (\mathbf {I}-\mathbf {A} (\mathbf {c})\big )\Big ), \end{aligned}$$

where $\mathbf {J}_{wfsic}=\mathbf {P}_w\mathbf {J}_w$ and $\mathbf {H}_{wfsic} = \mathbf {J}_{wfsic}^T\mathbf {J}_{wfsic}$, respectively. Finally, notice that in practice, we *never* calculate and store matrix multiplications of the form $\mathbf {W}\mathbf {X}$, for any matrix $\mathbf {X}\in \mathcal {R}^{N \times l}$. Essentially, the effect of this multiplication is a reduced size matrix of dimension $N_w \times l$, where $N_w$ is the number of non-zero elements in $\mathbf {W}$. In our implementation we used a grid such that $N_w/N=1/4$. Because $N_f=8$, the total cost of the algorithms is only increased by a factor of 2.

## Results

We have performed a number of experiments in order to report a comprehensive evaluation of the proposed algorithms. We present results for *3 Cases* of interest:

- *Case 1: Evaluation of pixel-based AAMs* We have already assessed the performance of all algorithms presented so far for the popular data set of LPFW. To verify these results, we report fitting performance for two challenging cross-database experiments on Helen (Le et al. 2012) and AFW (Zhu and Ramanan 2012). We emphasize that the faces of these databases contain significantly more shape and appearance variation than those of the training set of LFPW that all methods were trained on.
- *Case 2: Evaluation of SIFT-based AAMs* We report the fitting performance of the proposed algorithms when the appearance model was built using SIFT features for all three databases, and we focus on whether the proposed efficient weighted least-squares optimization of SIFT-based AAMs results in any loss in performance.
- *Case 3: Comparison with state-of-the-art* We present a comparison on both human and *animal* faces between the performance of the proposed algorithms against that of two of the best performing methods in literature, namely the Supervised Descent Method (SDM) of (Xiong and De la Torre 2013) and Chehra (Asthana et al. 2014). We also compare the performance of all methods considered in our experiments against the best possible fitting result achieved by an Oracle who knows the location of the landmarks in the test images and simply reconstructs them using the trained shape model.

Below, we summarize 3 main conclusions drawn from our experiments:

- *Conclusion 1* aFast-SIC and Fast-SIC feature the same performance both in terms of fitting accuracy and speed of convergence.
- *Conclusion 2* The part-based AAM (i.e. GN-DPM) built with SIFT features and fitted with E-POIC achieves essentially the same fitting accuracy as the same model fitted via aFast-SIC. To achieve this accuracy though, E-POIC requires about twice as many iterations. However, the cost per iteration for E-POIC is orders of magnitude smaller than the cost per iteration required for aFast-SIC.
- *Conclusion 3* Our two best performing methods, namely GN-DPMs built with SIFT features and fitted with aFast-SIC and E-POIC, outperform SDM and Chehra. However, SDM and Chehra converge faster.

We now provide the details of our experiments: All AAMs, including holistic and part-based (GN-DPMs), and pixel-based and SIFT-based, were trained on LFPW as described in Sect. 7. Landmark annotations based on the Multipie configuration for all databases (LFPW, Helen, AFW) are publicly available from the 300-W challenge (Sagonas et al. 2013). To fit all AAMs (both holistic and part-based), we used a multi-resolution approach with two levels. At the highest level the shape model has 15 shape eigenvectors and 400 appearance eigenvectors for all algorithms and AAMs. As for the subspace of the steepest descent images used in E-POIC, we used 1200 and 2400 components for pixel-based and SIFT-based AAMs, respectively.

It is worth noting that we used the efficient weighted least-squares optimization approach of Sect. 9 only for the case of SIFT features. In order to investigate whether such an approach results in loss in performance, we report results for Fast-SIC by optimizing over *all pixels* whilst we report results for aFast-SIC, E-POIC and POIC by optimizing over the points of the *sparse grid* described in Sect. 9. This setting is used for both AAMs and GN-DPMs. This means that if we exclude the cost for the SIFT extraction process, the aFast-SIC-SIFT, E-POIC-SIFT and POIC-SIFT algorithms have almost the same complexity as their pixel intensity counterparts (in particular the complexity is increased only by a factor of 2 as explained in Sect. 9).

Similarly to the experiment of Sect. 7, we initialized all algorithms using the bounding box of the face detector of Zhu and Ramanan (2012). To quantify performance, we produced the cumulative curves corresponding to the percentage of test images for which the normalized point-to-point error was less than a specific value. Note that for *cases 1 and 2*, we report performance for 68 points.

Regarding comparison with SDM and Chehra (*case 3*), we note that for the sake of a fair comparison we used the same implementation of SIFT that the authors of Xiong and De la Torre (2013) provide, although we used a reduced 8-dimensional SIFT representation as opposed to the 128-dimensional representation used in Xiong and De la Torre (2013). As our experiments have shown, this reduced representation seems to suffice for good performance and keeps the complexity of SIFT-based AAMs close to that of their pixel-based counterparts. Probably, the good performance can be attributed to the generative appearance model of the AAMs which can account for appearance variation. Finally, we carefully initialized both SDM and Chehra using the same face detector used for our AAMs, following the authors’ instructions, and we report performance on the 49 interior points because these are the points that the publicly available implementations of SDM and Chehra provide.

Figure 6 shows the results of *pixel-based* AAMs and GN-DPMs on Helen and AFW. Compared to LFPW, there is drop in performance for all methods because the faces of Helen and AFW are much more difficult to detect and fit. Nevertheless the relative difference in performance is similar, validating the conclusions of Sects. 7 and 8.1. Notably, the part-based representation and the translational motion model of GN-DPMs consistently outperform the holistic appearance models and the piecewise affine warp of AAMs.

Figure 7 shows the results obtained by fitting *SIFT-based* AAMs and GN-DPMs on LFPW, Helen and AFW. We may observe that (a) there is large boost in performance when SIFT features are used, (b) there is negligible difference in performance between Fast-SIC-SIFT which is optimized over *all pixels*, and aFast-SIC-SIFT which is optimized on a sparse grid, (c) E-POIC-SIFT on *a sparse grid* largely outperforms POIC-SIFT, and performs almost similarly to aFast-SIC-SIFT. Especially for the case of GN-DPMs, the difference in performance between aFast-SIC-SIFT and E-POIC-SIFT is almost negligible.

Additionally, Fig. 8 shows the convergence performance in terms of reduction of the average pt-pt error for a fixed number of iterations for all SIFT-based AAMs and algorithms. Note that similar results were obtained for the case of pixel-based AAMs but for brevity we present only the results for SIFT features which produce the best fitting performance. For this experiment, we used the test set of LPFW and a total of 50 iterations (25 for the lower and 25 for the higher level of our pyramid). As we may observe, the part-based formulation results in significantly faster error reduction. Additionally, Fast-SIC-SIFT and aFast-SIC-SIFT feature almost identical convergence performance. This result clearly illustrates that, compared to Fast-SIC, the aFast-SIC approximation essentially achieves the same performance in terms of both fitting accuracy and speed of convergence. From the same figure, we can observe that E-POIC-SIFT requires almost twice as many iterations at the lower level compared to Fast-SIC-SIFT and aFast-SIC-SIFT. Hence, although E-POIC-SIFT achieves very similar fitting performance to that of aFast-SIC-SIFT (especially for GN-DPMs), it also requires more iterations. However, the cost per iteration for each algorithm is significantly different. After ignoring the feature extraction step, E-POIC requires one matrix multiplication to calculate the update for the shape parameters which on an average laptop takes about 0.0003 s. To perform the necessary matrix multiplications to calculate the update for the shape and appearance parameters, aFast-SIC is approximately 100 times slower, while Fast-SIC is approximately 5 times slower than aFAST-SIC. It is worth noting that E-POIC is very attractive in terms of memory requirements as it requires storing only one matrix of size $N \times n$ in memory, while aFast-SIC additionally requires storing the appearance model and its gradients. This makes E-POIC particularly suitable for mobile applications.

Figure 9(a) shows the performance of our best performing GN-DPMs for different patch sizes $N_s$. It can be observed that the method is not too sensitive to patch size and that performance starts saturating already from $N_s=19$. Figure 9(b) compares performance for SIFT dimensionality equal to 8 and 128. We observe that there is no benefit in increasing SIFT dimensionality (and hence complexity). We attribute this to the flexibility of the generative appearance model employed by AAMs.

Figure 10 shows the comparison between our two best performing methods, namely GN-DPMs fitted via aFast-SIC-SIFT and E-POIC-SIFT and two state-of-the-art methods, namely SDM and Chehra. For this comparison, it is worth noting that we conducted experiments on human faces but also on *animal faces* For the former case, we followed our previous setting and trained aFast-SIC-SIFT and E-POIC-SIFT on about 800 images from LFPW. Note that SDM was trained on internal CMU data and Chehra on the whole LPFW, Helen and AFW data sets. As we may observe, the proposed methods outperform SDM on all three databases, and perform worse than Chehra only on the AFW data set. For the sake of a fairer comparison, we also provide the results of our implementation of SDM and Chehra, trained on LFPW. Finally, the later setting was repeated for our “Cats” data set which contains 1500 cat face images anotated with 42 landmarks (1000 images were used for training and 500 images for testing) selected from the Oxford pet data set Parkhi et al. (2012). Because large pose variations and facial hair are very common in cat faces, this data set is much more challenging than the ones containing human faces. As we may observe, compared to our implementations of SDM and Chehra, the proposed AAMs perform significantly better showing that AAMs can feature robust performance even when trained on relatively small data sets such as LFPW and very challenging data sets such as our “Cats” data set. We have to emphasize though that both SDM and Chehra require very few iterations to converge (about 5–6). Overall, these results clearly place the proposed methods in par with the state-of-the-art methods in face alignment. Finally, there is a very large performance gap between all methods and the best achievable result provided by the Oracle.

Finally, Fig. 11 shows some fitting examples of aFast-SIC-SIFT, E-POIC-SIFT, and POIC-SIFT on Helen. As we may observe aFast-SIC-SIFT and E-POIC-SIFT are significantly more robust and accurate than POIC-SIFT.

## Conclusions

We presented a framework for fitting AAMs to unconstrained images. Our focus was on robustness, fitting accuracy and efficiency. Toward these goals, we introduced several orthogonal contributions: First, we proposed a series of algorithms, perhaps the most notable of which are aFast-SIC-SIFT and more importantly E-POIC-SIFT. The former algorithm is relatively efficient, very accurate and very robust. The latter algorithm is very efficient, very accurate and, at the same time, notably robust. Secondly, we illustrated for the first time in literature the benefit of training AAMs in-the-wild. Thirdly, we introduced a part-based AAM combined with a translational motion model which is shown to largely outperform the holistic AAM based on piece-wise affine warps. Finally, we introduced a weighted least-squares formulation for the efficient fitting of SIFT-based AAMs. Via a number of experiments on the most popular unconstrained face databases (LPFW, Helen, AFW and Cats), we showed that E-POIC largely bridges the gap between exact and current approximate methods and performs comparably with state-of-the-art systems. Future work includes investigating how E-POIC can be extended for the case of regression-based techniques such as the one recently proposed in Tzimiropoulos (2015).

## Appendix

In this section we will derive (10) and (12). For convenience, we re-write the original optimization problem of (5)

31 $$\begin{aligned} \arg \min _{\varDelta \mathbf {p}, \varDelta \mathbf {c}} ||\mathbf {I}[\mathbf {p}]-\mathbf {A}_0 - \mathbf {A} \mathbf {c} - \mathbf {A} \varDelta \mathbf {c} - \mathbf {J} \varDelta \mathbf {p}||^2. \end{aligned}$$

To solve the above problem, our strategy is to optimize (31) firstly with respect to $\varDelta \mathbf {c}$, and then plug in the solution back to (31). Then, we can optimize (31) with respect to $\varDelta \mathbf {p}$. In particular, let us denote by $\mathbf {C}_1 = \mathbf {I}[\mathbf {p}] - \mathbf {A} ( \mathbf {c}) - \mathbf {J} \varDelta \mathbf {p}$. Then, we have

32 $$\begin{aligned} \arg \min _{\varDelta \mathbf {c}} (\mathbf {C}_1 - \mathbf {A}\varDelta \mathbf {c})^T(\mathbf {C}_1 - \mathbf {A} \varDelta \mathbf {c}). \end{aligned}$$

By setting the derivative of the above with respect to $\varDelta \mathbf {c}$ to $\mathbf {0}$, we readily obtain (10)

33 $$\begin{aligned} \varDelta \mathbf {c} = \mathbf {A}^T\mathbf {C}_1 = \mathbf {A}^T\Big (\mathbf {I}[\mathbf {p}] - \mathbf {A} \varDelta \mathbf {c} - \mathbf {J} \varDelta \mathbf {p}\Big ). \end{aligned}$$

Plugging the above to (31) and, after some straightforward mathematical manipulations, we get the following optimization problem for $\varDelta \mathbf {p}$

34 $$\begin{aligned} \arg \min _{\varDelta \mathbf {p}} \big (\mathbf {I}[\mathbf {p}] - \mathbf {J} \varDelta \mathbf {p}\big )^T\mathbf {P}\big (\mathbf {I}[\mathbf {p}] - \mathbf {J} \varDelta \mathbf {p}\big ), \end{aligned}$$

where $\mathbf {P} = \mathbf {E}- \mathbf {A}\mathbf {A}^T$. By setting the derivative of the above with respect to $\varDelta \mathbf {p}$ to $\mathbf {0}$, we readily obtain (12).

## Footnotes

1 This approach is different from the one used in Matthews and Baker (2004) which used composition. However, we found that which of the two ways is used for incorporating the global similarity transform makes little difference in the overall algorithms’ performance, and there is no approach that gives smaller error consistently for all images. We opted for using the “appending” version mainly because it simplifies mathematical notation. Please note that since we use the same approach for all algorithms, we ensure that comparisons are fair.

2 For a vector $\mathbf {x}$, we use the notation $||\mathbf {x}||_{\mathbf {P}_A}^2$ to denote the weighted norm $\mathbf {x}^T\mathbf {P}_A\mathbf {x}$.
